# Editorial: Breast milk and passive immunity during the COVID-19 pandemic

**DOI:** 10.3389/fnut.2023.1155901

**Published:** 2023-02-22

**Authors:** Veronique Demers-Mathieu

**Affiliations:** Department of Molecular Immunology, Exagen Inc., Vista, CA, United States

**Keywords:** neutralizing antibody, mucosal immunity, breast milk antibodies, mRNA-based vaccine, lactating women, pregnant women, serum antibodies, immunogenicity

Neonates are born with an immature immune system, including a lack of IgG and secretory IgA (SIgA) production by plasma cells. Thus, newborns rely on the passive transfer of antibodies *via* the placenta (only IgG) and breast milk (80–90% SIgA/IgA, 5% IgG, and 10–15% IgM) to protect them against severe acute respiratory syndrome coronavirus 2 (SARS-CoV-2) infection during the first 2 to 6 months of postnatal age. This editorial presents 10 contributing articles on the Research Topic “*Breast Milk and Passive Immunity during the COVID-19 Pandemic*.” First, we describe the passive immunity from the placenta to the fetus after mRNA COVID-19 vaccination. Second, we evaluate the risk of transplacental transmission of SARS-CoV-2 to the fetus. Third, we elucidate that breast milk is not a vector of viral SARS-CoV-2 that can infect breastfed infants. Fourth, we discuss the maternal antibody response specific to SARS-CoV-2 after the two mRNA COVID-19 vaccine and the booster dose. Fifth, we report the safety of the mRNA COVID-19 vaccine and booster shot during breastfeeding. Lastly, we describe the relationship between maternal stress and antibody response in lactating mothers.

## Passive immunity from the placenta to the fetus after mRNA COVID-19 vaccination

**Figure 1:**
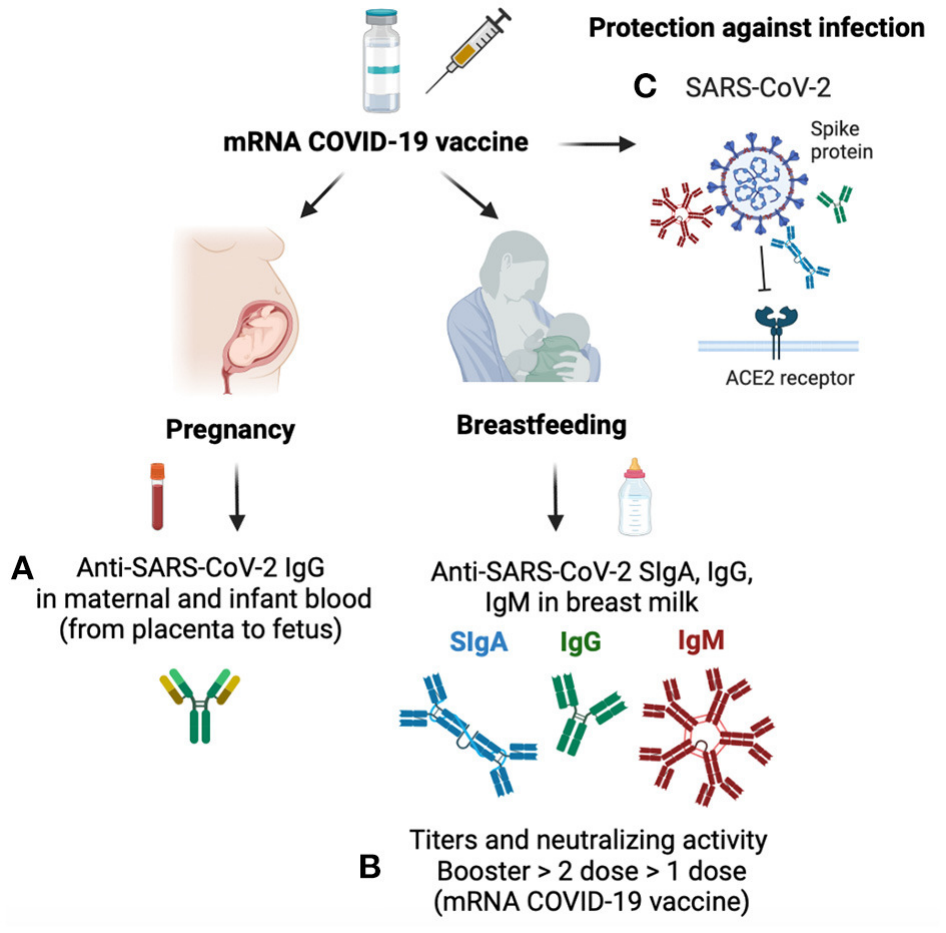
Mechanisms of antibody transfer during pregnancy and breastfeeding after mRNA COVID-19 vaccine. **(A)** Anti-SARS-CoV-2 IgG is transferred from the placenta to the fetus and is present in cord blood and infant blood from mothers vaccinated while pregnant. **(B)** Breast milk contains anti-SARS-CoV-2 IgG, IgA, and IgM after the first and second mRNA COVID-19 vaccines. Neutralizing activity and titer of anti-SARS-CoV-2 antibodies are higher after the booster (third dose) than after the second dose of the mRNA COVID-19 vaccine in breast milk, while only one dose has the lowest neutralizing capacity and antibody titers. **(C)** Anti-SARS-CoV-2 antibodies bind to the spike protein of SARS-CoV-2, which block the viral attachment to the angiotensin-converting enzyme (ACE) receptor and provide protection against COVID-19 infection in mothers and their breastfed infants.

IgG is the only isotype passively transferred from the placenta to the fetus during pregnancy. Anti-SARS-CoV-2 IgG was present in cord blood and infants' blood from mothers vaccinated while pregnant, confirming the transfer of anti-SARS-CoV-2 IgG *via* the placenta to the fetal bloodstream (Hunagund et al.; Figure 1A). In contrast, serum anti-SARS-CoV-2 IgG was absent in infants born from mothers vaccinated after pregnancy, which could reduce their protection against COVID-19 infection.

## Low risk of transplacental transmission of SARS-CoV-2 to the fetus

Most neonates born from COVID-19-infected mothers did not test positive for COVID-19, while few cases of newborns tested positive and presented early-onset symptoms (Rad et al.). Whether newborns with positive COVID-19 are due to the transplacental transmission of SARS-CoV-2 or infection after delivery is still not well-understood. The placenta provides a protective barrier to the fetus against maternal infections. However, vertical transmission of SARS-CoV-2 from the placenta to the fetus can happen when the virus-induce apoptosis and vascular damage in the placenta. SARS-CoV-2 can spread in maternal endothelium into fetal capillaries and then be aspirated through amniotic fluids. The presence of SARS-CoV-2 in blood samples from newborns was ~1%, suggesting the risk of transplacental transmission of COVID-19 to the fetus is low.

## Breast milk is not a vector of viral SARS-CoV-2 that can infect breastfed infants

A few articles have reported the detection of low titers of viral SARS-CoV-2 RNA in breast milk samples. Lactating mothers and health professionals have been worried about the potential transfer of viral SARS-CoV-2 from breast milk to the infant. To confirm the safety of breastfeeding during maternal COVID-19 infection, breast milk was collected before and after washing the breast skin in lactating mothers with COVID-19 infection. Some breast skin swabs collected before washing the breast detected SARS-CoV-2 RNA in milk samples, while SARS-CoV-2 RNA was absent in all milk samples after washing the breast skin (Pace et al.). Breast skin contaminated with SARS-CoV-2 was associated with the presence of maternal caught and other family members in the household with COVID-19 infection. These findings explain why some breast milk samples in previous studies have detected positive results for SARS-CoV-2 RNA using RT-qPCR. Breast milk is likely not a potential source of viral RNA SARS-CoV-2 when mothers wash their breast skin before breastfeeding.

## Maternal antibody response specific to SARS-CoV-2 during COVID-19 vaccine

After two mRNA COVID-19 vaccine doses, lactating mothers had detectable anti-SARS-CoV-2 IgG1, IgA, and IgM in serum samples, with an increase in all three isotypes after the second dose, especially IgG1 levels (Yeo et al.). After the second vaccine, all mothers had detectable anti-SARS-CoV-2 IgG1 and IgA in breast milk, whereas IgM was present in 87% of milk samples. Neutralizing antibodies increased after the second dose in serum and breast milk compared to the first dose (Figure 1B). The rapid increase of IgG after the second dose correlated with the specific B lymphocyte memory that prime a faster response with higher antibody levels. In contrast, IgA levels remained constant between the two first doses of the COVID-19 vaccine. Infants breastfed from vaccinated mothers did not have detectable neutralizing antibodies or vaccine mRNA in their serum.

The mRNA COVID-19 booster (total of three doses of mRNA vaccine) elevated antibody secretion in lactating mothers. The titers of IgG and IgA specific to SARS-CoV-2 were higher in breast milk after a third booster dose of mRNA COVID-19 than the peak after the first and second vaccines (Bender et al.; Figure 1B). The neutralizing capacity of breast milk antibodies produced during the third mRNA COVID-19 booster shot was higher than those with the second COVID-19 vaccine (pre-booster sample). Neutralizing activity of breast milk antibodies correlated with serum antibodies in mothers with the booster dose. These findings support the current guidance that all pregnant or lactating mothers should receive the mRNA COVID-19 booster dose to provide an optimal mucosal response to the mothers. Increased antibody secretion in vaccinated mothers during lactation could promote neonatal mucosal immunity *via* ingestion of breast milk IgA, IgG, and IgM. Anti-SARS-CoV-2 antibodies bind to the spike protein of SARS-CoV-2, which block the viral attachment to the angiotensin-converting enzyme (ACE) receptor and provide protection against COVID-19 infection (Figure 1C).

Like the mRNA vaccine, vector-based vaccines stimulated antibody response in lactating mothers. Paired longitudinal samples taken at 45 and 120 days after the second dose of COVID-19 vector-based vaccines showed that IgG levels waned over time in breast milk, while IgA levels remained stable in 100% of lactating women (Longueira et al.). A slight reduction of IgA titers in serum relative to paired breast milk samples was detected 120 days after the second vector vaccine dose, suggesting a more sustained IgA production in mucosal secretion. In contrast, IgG levels in serum and breast milk (paired samples) decreased from 45 to 120 days after the second vaccine.

## The mRNA COVID-19 vaccine is safe during breastfeeding

Some lactating mothers are hesitant to receive a COVID-19 vaccine due to the lack of knowledge on the immunogenicity of mRNA-based vaccines on nursing infants, as lactating mothers were excluded from initial clinical trials of mRNA vaccination. Paired blood and milk samples from lactating mothers and their infants were collected after the maternal mRNA vaccine (2 doses) to evaluate the immunogenicity of the mRNA molecule. Severe side effects were absent in infants breastfed from mothers vaccinated with mRNA COVID-19 (Golan et al.). Vaccine-related PEGylated protein concentrations did not increase in breast milk after COVID-19 vaccination. These results suggest that the mRNA-COVID-19 vaccine administered in lactating women did not lead to detectable immunogenicity in the infant's blood. In addition, low levels of intact mRNA vaccine were detected in maternal serum and breast milk samples, while infants' serum had no trace of mRNA molecule or serological evidence of infant sensitization (Yeo et al.). These findings confirm the safety of continuing breastfeeding during maternal mRNA COVID-19 vaccination.

A systemic review article demonstrated that lactating women receiving 2 doses of the mRNA COVID-19 vaccine are safe for them and their breastfed infants (Muyldermans et al.). Another mini-review article assessed that the safety and efficacy of the developed mRNA COVID-19 vaccines were comparable between pregnant, lactating, and non-pregnant women (Laguila Altoé et al.). The administration of mRNA COVID-19 vaccination in these groups promoted the production of neutralizing antibodies against SARS-CoV-2 in mothers and passive immunity in their infants.

## Maternal stress and antibody response in lactating mothers

Antibody response is influenced by several factors related to maternal background and confounding factors, including psychological stress. Maternal stress could be elevated by stressful events, including the COVID-19 pandemic. Interestingly, the stress levels of lactating women were comparable between pre-pandemic and during the pandemic COVID-19 (Juncker et al.). However, maternal lifetime stressors were negatively correlated with breast milk IgA specific to SARS-CoV-2. Breastfed infants of mothers with high chronic stress levels might ingest lower breast milk SIgA/IgA.

## Conclusion and perspective

This Research Topic in Frontiers in Nutritional Immunology provided new knowledge on the activation of antibody response during mRNA COVID-19 vaccine and booster dose in pregnant and lactating mothers and confirmed its safety for their infants. Additional investigations are required to identify the mechanisms of antibody protection when infants ingest breast milk with neutralizing antibodies against SARS-CoV-2. Finally, more studies are needed to reveal which maternal factors (including genetics, nutrition, preexisting immunity, and health conditions) enhance antibody responses during vaccination.

## Author contributions

VD-M wrote the first draft of the manuscript, sections of the editorial article, revised, read, and approved the submitted version.

